# Oroxylin A inhibits colitis by inactivating NLRP3 inflammasome

**DOI:** 10.18632/oncotarget.19440

**Published:** 2017-07-22

**Authors:** Wei Zhou, Xiuting Liu, Xin Zhang, Jingjing Tang, Zhiyu Li, Qing Wang, Rong Hu

**Affiliations:** ^1^ State Key Laboratory of Natural Medicines, Department of Physiology, China Pharmaceutical University, Nanjing 210009, China; ^2^ Department of Medicinal Chemistry, China Pharmaceutical University, Nanjing 210009, China; ^3^ Department of Neurosurgery, Wuxi Second Hospital Affiliated Nanjing Medical University, Wuxi 214002, China

**Keywords:** NLRP3 inflammasome, inflammatory bowel disease, oroxylin A, NF-κB, DSS-induced colitis

## Abstract

NLRP3 inflammasome is a novel therapeutic target for inflammatory bowel disease (IBD). The aim of this study was to investigate the anti-inflammatory effect of a bioactive flavonoid—oroxylin A on the treatment of dextran sulfate sodium (DSS)-induced murine colitis via targeting NLRP3 inflammasome. In this study, we found that oroxylin A attenuated experimental colitis in mice, including loss of body weights, shortening of the colon lengths and infiltration of inflammatory cells. The production of IL-1β, IL-6 and TNF-α in colon was also markedly reduced by oroxylin A. Moreover, oroxylin A significantly decreased the expression of NLRP3 in intestinal mucosal tissue. In addition, NLRP3-/- mice were observably protected from DSS-induced acute colitis, and oroxylin A treatment had no effects on attenuating inflammation in NLRP3-/- mice. Further study found that the activation of NLRP3 inflammasome was dose-dependently inhibited by oroxylin A in both THP-Ms and BMDMs, followed by decrease in the cleavage of caspase-1 and secretion of IL-1β. This inhibitory effect of oroxylin A was due to restraint of the NLRP3 protein expression and the inflammasome formation in macrophages. Furthermore, the reduction of NLRP3 protein expression by oroxylin A was dependent on the inhibition of NF-κB p65 expression and nuclear translocation. Besides, oroxylin A directly suppressed the ASC speck formation and the inflammasome assembly which in turn restrained the activation of NLRP3 inflammasome. Our findings demonstrated that oroxylin A inhibited NLRP3 inflammasome activation and could potentially be used for the treatment of IBD.

## INTRODUCTION

Inflammatory bowel disease (IBD) is chronic inflammatory bowel diseases characterized by repeated abdominal pain and diarrhea [1]. IBD, including Crohn's disease and ulcerative colitis, poses a health risk in developed countries [2], with more than one million sufferers in the United States [3]. The symptoms of the patients with IBD are mostly caused by the decreased efficiency of the epithelial barrier and dysregulated immune response to commensal microflora [4]. Besides, IBD is characterized by a massive infiltration of neutrophils, lymphocytes, and monocytes in the intestinal tract. Upon activation, these immune cells produce reactive metabolites of oxygen and proinflammatory cytokines which strongly correlate with the grade of IBD [5]. However, the exact cause of this disease remains largely unknown.

NLRP3, a best-characterized member of the NOD-like receptor (NLR) families, contains a pyrin domain at the N-terminus which interacts with an adaptor protein, the apoptosis-associated speck-like protein (ASC). Once activated, NLRP3, ASC and pro-caspase 1 constitute the multiprotein complex, the NLRP3 inflammasome, which then activates the pro-caspase 1, and the cleaved-caspase-1 will in turn produce the biological active forms of IL-1β and IL-18 [6]. NLRP3 has been shown to play a crucial role in the IBD development. Villani AC et al. suggested that the mutations in NLRP3 contribute to the susceptibility of Crohn's disease [7]. Bauer C et al. reported that NLRP3-deficient mice have attenuated colitis and reduced mortality which due to increased immune cells [8]. Study by Guo W et al. also demonstrated that the small molecule andrographolide (Andro) ameliorates mice against colitis-associated cancer through inhibiting NLRP3 inflammasome activation [9]. These findings indicate that negative regulation of NLRP3 can be a promising approach for treating IBD. To investigate this disease in murine model, dextran sulfate sodium (DSS)-induced mouse colitis model is introduced. Oral administration of DSS will destroy colonic mucosal permeability, and then lead to mononuclear cell invasion in the colon tissue and trigger inflammation [10, 11]. Moreover, NLRP3 inflammasome has been reported to play an important role in colitis severity and inflammatory response in the DSS-induced model [12].

Oroxylin A is a natural flavonoid that is one of the major constituents of the root of *Scutellaria baicalensis*, which exhibits several beneficial effects including cytoprotection, anti-inflammation and anti-cancer activities [13–15]. Recent evidences indicated that oroxylin A can inhibit LPS-induced cyclooxygenase-2 (COX-2) and inducible Nitric Oxide Synthase (iNOS) expressions in macrophages via regulation of NF-κB pathway [16]. Furthermore, the repressive effect of oroxylin A on NF-κB signaling results in the recruitment of additional immune cells and secretion of inflammatory cytokines [13]. In recent years, oroxylin A has been reported as a potential anti-inflammation agent [15]. Several articles indicated that oroxylin A significantly attenuates LPS-induced lung injuries and increases survival rate of the animals through inhibition of NF-κB pathway and release of cytokines, including cytoplasmic NF-κB-targeting HMGB1, NO and TNF-α [13]. We previously found that oroxylin A inhibits inflammation-associated proliferation of tumor cells by modulating the IL-6/STAT3 signaling and NF-κB signaling pathways which are involved in inflammation-induced cancer initiation and progression [14, 17]. Therefore, oroxylin A is suggested to be a potential agent for the treatment of inflammatory bowel diseases. In this study, we found that oroxylin A remarkably attenuated the symptoms of murine colitis and decreased the activation of NLRP3 inflammasome induced by DSS in colitis model. *In vitro* study showed that the treatment with oroxylin A reduced NLRP3 protein expression and inflammasome formation, resulting in the decrease of cleaved-caspase-1 and active IL-1β levels in macrophages.

## RESULTS

### Dietary administration of oroxylin A attenuated DSS-induced chronic colitis through NLRP3 inflammasome

**Figure 1:**
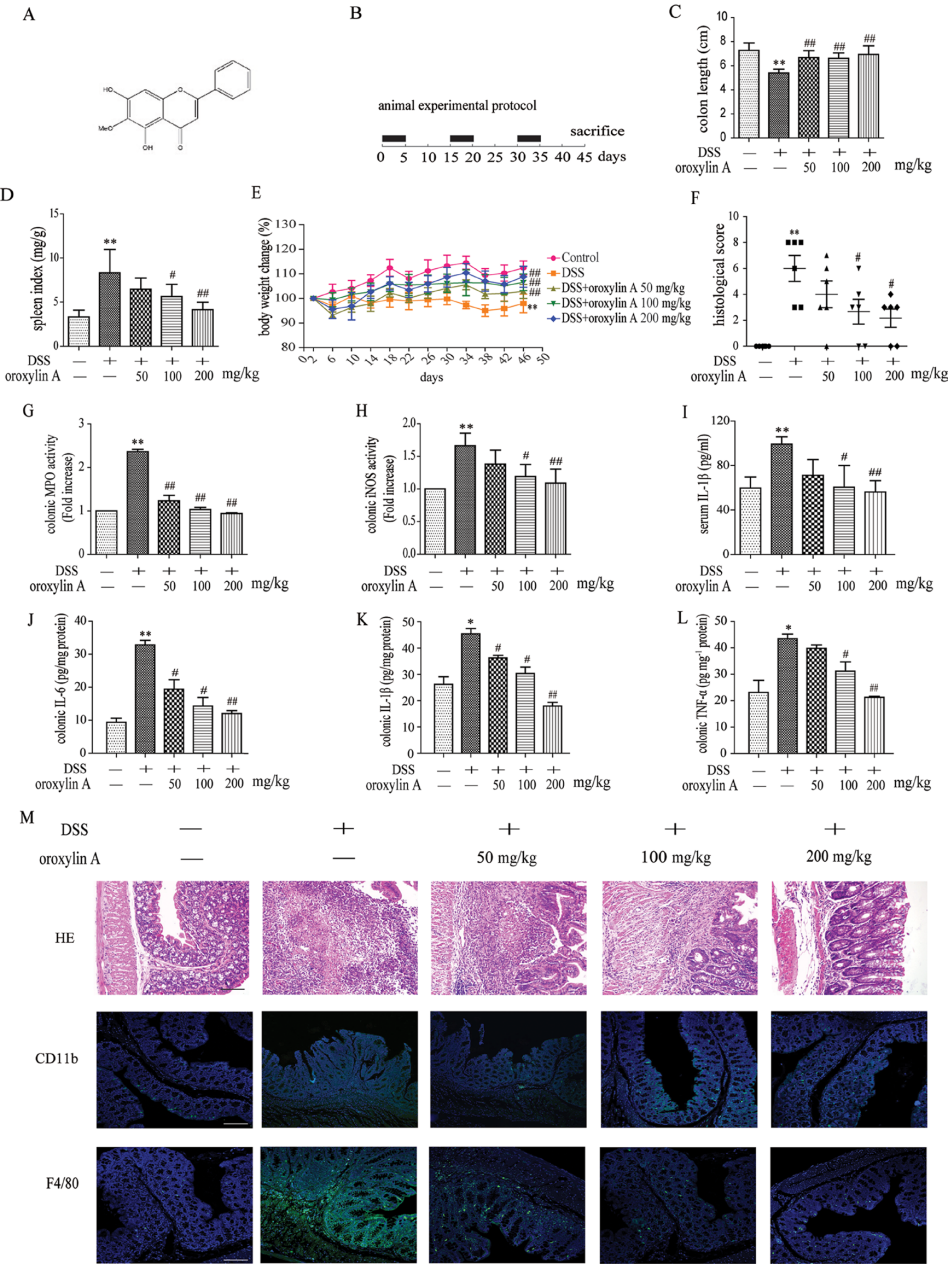
Dietary administration of oroxylin A attenuated DSS-induced chronic colitis (**A**) Molecular structure of oroxylin A (C_16_H_12_O_5_. MW: 284.27). (**B**) The experimental protocol for the treatment with oroxylin A in chronic colitis model. (**C**, **D**) The lengths of colons and spleen indexes of each group were measured (*n* = 10). (**E**) The changes of body weight in each group during the disease process (*n* = 10). (**F**) Histopathological scores of each group were determined (*n* = 6). (**G**) MPO and (**H**) iNOS activities in the colonic tissues were detected (n = 6). (**I**) The secretion of IL-1β in serum was determined by ELISA. (**J**–**L**) The protein levels of IL-1β, TNF-α and IL-6 in colonic tissue were determined. (**M**) Serial sections of colon tissues were stained with HE. Sections of colonic tissue were immunostained for DAPI (blue) and CD11b-FITC (green) or F4/80-FITC (green) (scale bar, 100 μm). Data were shown as means ± SD (*n* = 3). Statistical analysis was performed using one-way ANOVA coupled with a post hoc test. Significant differences were indicated as *P < 0.05 vs. control group (^#^*P* < 0.05 vs. DSS group); **P < 0.01 vs. control group (^##^*P* < 0.01 vs. DSS group).

DSS-induced colitis is the most widely used animal model for preclinical testing of IBD [18]. To investigate the anti-inflammatory effects of oroxylin A on experimental colitis *in vivo*, we established the chronic experimental colitis model in C57BL/6 mice (Figure 1B). Damage of crypts, infiltration of mononuclear cells, and severe mucosal destruction were observed in the colon specimens of DSS-treated mice. Oroxylin A markedly attenuated the loss of body weight, splenomegaly and colonic shortening induced by DSS during the colitis progression (Figure 1C–1E). The results from standard pathological test showed that oroxylin A exhibited notable protective effects against intestine inflammation (Figure 1F and Figure 1M). Moreover, oroxylin A significantly attenuated hyperactivated MPO and iNOS induced by DSS in colonic tissues (Figure 1G and 1H). It also remarkably inhibited the elevated production of IL-1β in the serum (Figure 1I). Besides, it inhibited pro-inflammatory cytokines production, including IL-6, IL-1β and TNF-α, in colon tissues from DSS-treated mice (Figure 1J–1L). Additionally, oroxylin A significantly abolished the distribution of CD11b+ inflammatory cells and F4/80+ macrophages in DSS-treated colon tissues (Figure 1M). Taken together, these results showed that oroxylin A ameliorated DSS-induced chronic colitis.

**Figure 2:**
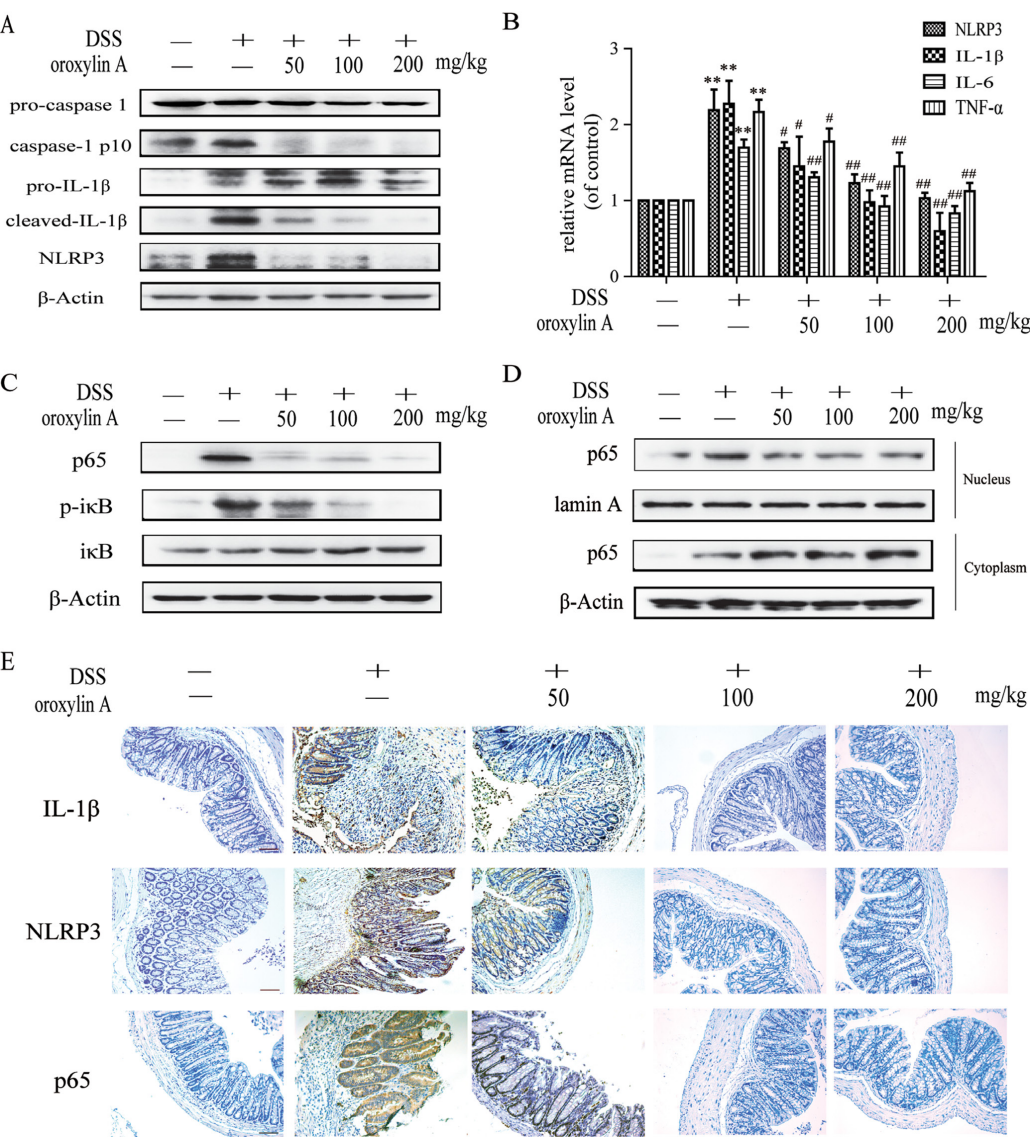
Oroxylin A suppressed the activation of NLRP3 inflammasome in DSS-induced chronic colitis (**A**) The protein levels of cleaved-caspase-1 (caspase-1 p10), cleaved-IL-1β and NLRP3 in colonic tissues were detected by western blot. (**B**) The mRNA levels of NLRP3, IL-1β and IL-6 in colonic homogenate were determined by real-time RT-PCR. (**C**) The NF-κB p65, IκBα phosphorylation in colonic tissues were detected by western blot. (**D**) The relative expressions of cytoplastic protein and nuclear protein NF-κB p65 in colonic tissues were assessed by western blot. (**E**) Immunohistochemistry of IL-1β, NLRP3 and p65 in colonic tissues of each group was measured. Brown colored is positive (scale bar, 100 μm). Data were shown as means ± SD (*n* = 3). Statistical analysis was performed using one-way ANOVA coupled with a post hoc test. Significant differences were indicated as * *P* < 0.05 vs. control group (^#^*P* < 0.05 vs. DSS group); ** *P* < 0.01 vs. control group (^##^*P* < 0.01 vs. DSS group).

It is known that NLRP3 inflammasome plays a key role in the DSS-induced murine colitis [12]. Consistent with the previous results, oroxylin A could significantly relieve acute colitis in mice. As shown in Figure 2A, oroxylin A exhibited a significant inhibition on protein levels of cleaved-IL-1β, cleaved-caspase-1 (caspase-1 p10) and NLRP3 in colon tissues, suggesting that the protective effects of oroxylin A were ascribed to the down-regulation of NLRP3 inflammasome. Furthermore, oroxylin A significantly inhibited DSS-increased mRNA levels of NLRP3, IL-1β, IL-6 and TNF-α (Figure 2B), which prompted that the inhibition of NLRP3 mRNA might be mediated by its upstream NF-κB pathway [19]. Indeed, the oroxylin A could significantly repress the increased NF-κB expression and IκBα phosphorylation induced by DSS treatment (Figure 2C). We also observed that nuclear translocation of p65 was dramatically restrained by oroxylin A treatment (Figure 2D). Furthermore, the inhibition on the expressions of NLRP3, IL-1β and p65 by oroxylin A was verified by immunohistochemical assays (Figure 2E). Taken together, these findings suggested that oroxylin A markedly suppressed NLRP3 inflammasome activation through inhibiting NF-κB pathway *in vivo*.

### Oroxylin A ameliorated DSS-induced acute colitis via NLRP3 inflammasome

**Figure 3:**
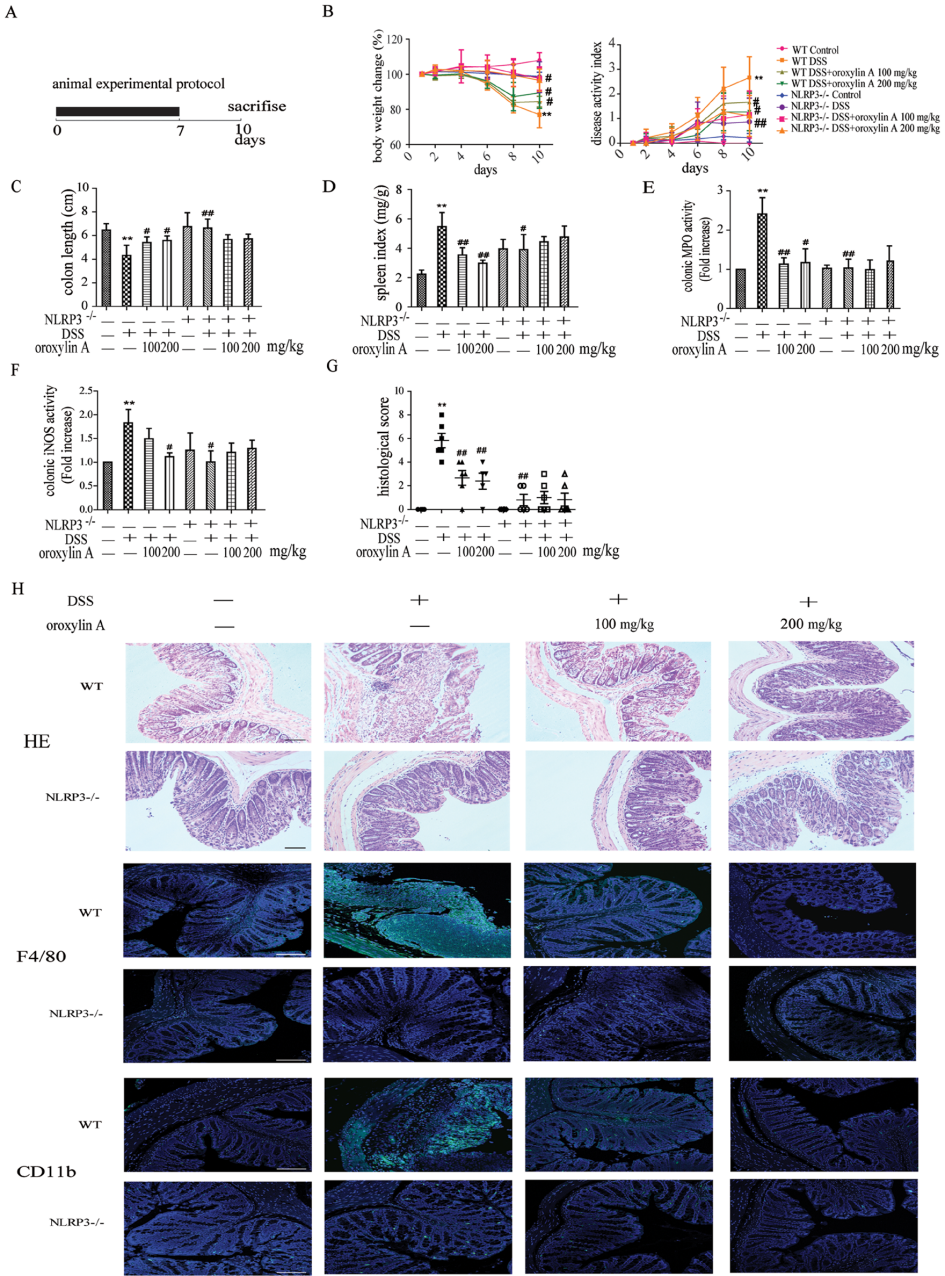
Oroxylin A ameliorated the symptoms of DSS-induced acute colitis via NLRP3 inflammasome (**A**) The experimental protocol for the treatment with oroxylin A in acute colitis model. (**B**) The changes of body weight in each group during the disease process. Disease activity index (DAI) of each group was evaluated. (**C**, **D**) The lengths of colons and spleen indexes of each group were measured (*n* = 6). (**E**) MPO and (**F**) iNOS activities in the colonic tissues were detected (*n* = 6). (**G**) Histopathological scores of each group were determined (n = 6). (**H**) Serial sections of colon tissues were stained with HE. Sections of colonic tissue were immunostained for DAPI (blue) and CD11b-FITC (green) or F4/80-FITC (green) (scale bar, 100 μm). Data were shown as means ± SD (n = 3). Statistical analysis was performed using one-way ANOVA coupled with a post hoc test. Significant differences were indicated as *P < 0.05 vs. control group (^#^*P* < 0.05 vs. DSS group); **P < 0.01 vs. control group (^##^*P* < 0.01 vs. DSS group).

To further investigate the anti-inflammatory effects of oroxylin A in acute colitis, oroxylin A were daily administered to mice during DSS-induced acute colitis (Figure 3A). Compared with DSS group, the reduction of body weight was markely attenuated by oroxylin A administion during the colitis progression (Figure 3B). Moreover, oroxylin A evidently ameliorated the disease activity index (DAI), which reflected the severity of the colitis (Figure 3B). The colonic shortening and splenomegalia were also observably improved by oroxylin A treatment (Figure 3C and Figure 3D). Besides, the DSS-induced hyperactivated MPO and iNOS were significantly reduced in oroxylin A group (Figure 3E and Figure 3F). Histological analysis revealed that DSS caused distortion of crypts, infiltration of mononuclear cells and severe mucosal damage in the colon tissues. However, oroxylin A effectively improved these symptoms (Figure 3G and 3H). We also examined CD11b+ inflammatory cells and F4/80+ macrophages infiltration in colon tissues. The results showed that oroxylin A observably decreased the inflammatory cells and macrophages infiltration in acute colitis model in WT mice (Figure 3H).

NLRP3 inflammasome has been reported to play a critical role in DSS-induced acute colitis model [7]. To further confirm the role of NLRP3 inflammasome in oroxylin A-mediated protective effects, NLRP3-/- mice were used. Compared with WT mice, deletion of NLRP3 significantly attenuated the severity of symptoms in DSS-induced colitis, with decreased weight loss, colon shortening and spleen enlargement (Figure 3B, Figure 3C and Figure 3D). The DSS-increased MPO and iNOS levels were decreased after oroxylin A treatment in WT mice, but were not in NLRP3-/- mice (Figure 3E and 3F). In addition, HE staining and histological analysis revealed a remarkable improvement of pathological changes in NLRP3-/- mice compared with the WT during colitis progression, including reduction of necrosis, mucosal damage and less distribution of inflammatory cells (Figure 3G and 3H). Besides, deletion of NLRP3 remarkably decreased the infiltration of CD11b+ inflammatory cells and F4/80+ macrophages (Figure 3H). Moreover, oroxylin A did not relieve NLRP3-/- mice from the severity of colitis symptoms (Figure 3H). These findings indicated that NLRP3 inflammasome played an important role in DSS-induced acute colitis, and its inhibition was critical to the protective effects of oroxylin A in DSS-treated mice.

**Figure 4:**
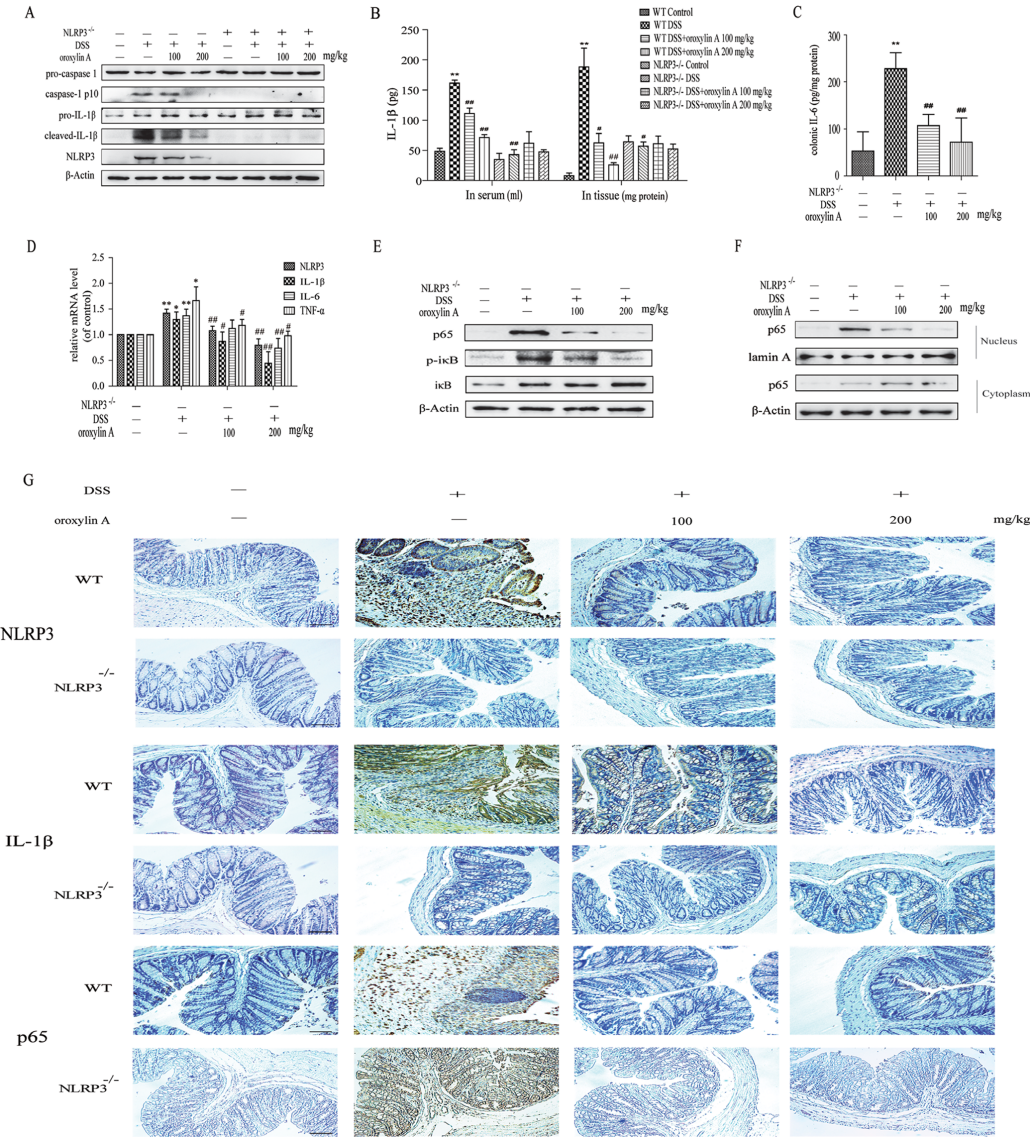
Oroxylin A down-regulated the activation of NLRP3 inflammasome in DSS-induced acute colitis (**A**) The protein levels of cleaved-caspase-1 (caspase-1 p10), cleaved-IL-1β and NLRP3 in colonic tissues were detected by western blot. (**B**) The serum IL-1β, colonic IL-1β and (**C**) colonic IL-6 were determined by ELISA. (**D**) The mRNA levels of NLRP3, IL-1β, IL-6 and TNF-α in colonic tissue were determined by real-time RT-PCR. (**E**) The NF-κB p65 and IκBα phosphorylation in colonic tissues were detected by western blot. (**F**) The relative expressions of cytoplastic protein NF-κB p65 and nuclear protein NF-κB p65 in colonic tissues were assessed by western blot. (**G**) Immunohistochemistry of NLRP3, IL-1β and p65 in colonic tissues of each group was measured. Brown colored is positive (scale bar, 100 μm). Data were shown as means ± SD (*n* = 3). Statistical analysis was performed using one-way ANOVA coupled with a post hoc test. Significant differences were indicated as *P < 0.05 vs. control group (^#^*P* < 0.05 vs. DSS group); **P < 0.01 vs. control group (^##^*P* < 0.01 vs. DSS group).

To further explore the mechanism of oroxylin A-mediated protection from colitis, we examined the expression of NLRP3 inflammasome in acute colitis. As shown in Figure 4A, oroxylin A exhibited a significant inhibition on the elevated expressions of NLRP3, cleaved-caspase-1 (caspase-1 p10) and cleaved-IL-1β. Furthermore, we examined the IL-1β levels in serum and colonic tissues of WT mice. As shown in Figure 4B, oroxylin A exhibited a significant inhibition on DSS-induced IL-1β levels in serum and colon in a dose-dependent manner. The inflammatory cytokines IL-6 induced by DSS was also decreased by oroxylin A treatment (Figure 4C). Moreover, immunohistochemical results revealed that DSS-induced increases of NLRP3 and IL-1β levels were markedly suppressed by oroxylin A in WT mice (Figure 4G). The results of western blot and immunohistochemical showed an undetectable level of NLRP3 and cleaved-IL-1β expression in colonic tissue of NLRP3-/- mice (Figure 4A and 4G). Besides, the IL-1β levels in serum and colon were not altered in NLRP3-/- mice (Figure 4B). In addition, oroxylin A treatment significantly inhibited the elevated mRNA expressions of NLRP3, IL-1β, IL-6 and TNF-α in DSS-treated WT mice, which illustrated that the adverse effects of oroxylin A might be mediated by the NF-κB pathway (Figure 4D). As expected, the results of western blot and immunohistochemical revealed that the protein level of p65 was suppressed by oroxylin A in a concentration-dependent manner (Figure 4E and 4G). In addition, the nuclear translocation of NF-κB p65 was also notably suppressed after oroxylin A treatment (Figure 4F). Collectively, these data demonstrated that oroxylin A markedly reduced DSS-induced colitis through inhibiting NLRP3 inflammasome.

### Oroxylin A inhibited NLRP3 inflammasome activation in THP-Ms and BMDMs

**Figure 5:**
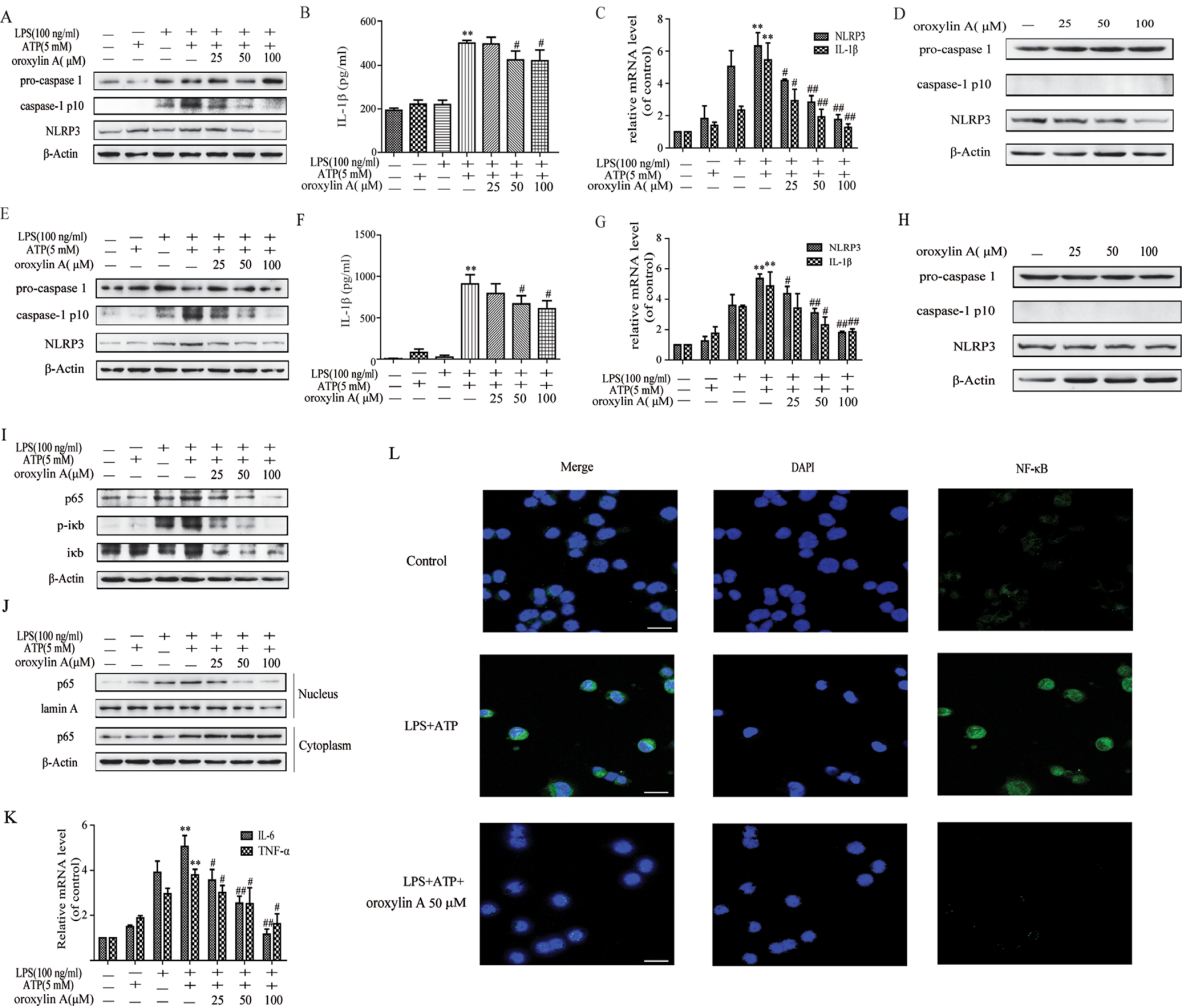
Oroxylin A inhibited NLRP3 inflammasome activation in THP-Ms and BMDMs (**A**) THP-Ms were stimulated with LPS (100 μM) and ATP (5 mM), then treated with indicated concentrations of oroxylin A for 12 h. The expressions of NLRP3, pro-caspase 1 and cleaved-caspase-1 (caspase-1 p10) proteins were assessed by western blot. (**B**) The secretion of IL-1β in supernatant was determined by ELISA. (**C**) The mRNA levels of NLRP3 and IL-1β were detected by real-time RT-PCR. (**D**) THP-Ms were treated with oroxylin A for 12 h at indicated doses, then protein levels of NLRP3, pro-caspase 1 and cleaved-caspase-1 (caspase-1 p10) were assessed by western blot. (**E**) BMDMs were stimulated with LPS (100 μM) and ATP (5 mM), then treated with oroxylin A for 12 h. The expressions of NLRP3, pro-caspase-1 and cleaved-caspase-1 (caspase-1 p10) proteins were assessed by western blot. (**F**) The amount of IL-1β in supernatant was determined by ELISA. (**G**) The expressions of NLRP3 and IL-1β mRNA in BMDMs were detected by real-time RT-PCR. (**H**) BMDMs were treated with oroxylin A for 12 h at indicated doses, the protein levels of NLRP3 and pro-caspase 1 were assessed by western blot. (**I**) The protein expressions of NF-κB p65, p-IκB and IκB were measured by western blot in THP-Ms. (**J**) The relative expressions of cytoplastic protein NF-κB p65 and nuclear protein NF-κB p65 in THP-Ms were assessed by western blot. (**K**) The mRNA expressions of IL-6 and TNF-α in THP-Ms were detected by real-time RT-PCR. (**L**) Immunofluorescence was performed to analyze NF-κB p65 nuclear translocation (scale bar, 20 μm). Data were shown as means ± SD (*n* = 3). Statistical analysis was performed using one-way ANOVA coupled with a post hoc test. Significant differences were indicated as *P < 0.05 vs. control group (^#^*P* < 0.05 vs. DSS group); **P < 0.01 vs. control group (^##^*P* < 0.01 vs. DSS group).

NLRP3 inflammasome activation is a two-step process. The first step requires surface molecules derived from microorganisms or other inflammatory factors (e.g. LPS) as first signal to activate NF-κB, followed by increased NLRP3 expression, as well as pro-IL-1β and pro-IL-18 protein production. The second signal of the extracellular ATP or bacterial toxins directly stimulates NLRP3 inflammasome assembly, which leads to caspase-1 activation, thereby secreting IL-1β and IL-18 [20]. To determine the effect of oroxylin A on NLRP3 inflammasome activation *in vitro*, LPS-primed THP-Ms were treated with oroxylin A after ATP challenge. NLRP3-dependent caspase-1 activation and the secretion of IL-1β were dose-dependently inhibited by oroxylin A (Figure 5A and Figure 5B). Moreover, the mRNA expressions of NLRP3 and IL-1β were also inhibited by oroxylin A in a dose-dependent manner, suggesting that oroxylin A might inhibit NLRP3 inflammasome activation via affecting the priming step (Figure 5C). Consistently, oroxylin A effectively reduced the protein expression of NLRP3 in THP-Ms without inflammatory stimulation (Figure 5D). These findings suggested that oroxylin A inhibited NLRP3 inflammasome activation and subsequent IL-1β secretion in THP-Ms.

To further corroborate the inhibitory effect of oroxylin A on NLRP3 inflammasome, we evaluated oroxylin A's effects using another cell model, BMDMs, which could reflect the physiological characteristics of NLRP3 inflammasome *in vitro* [20, 21]. NLRP3 inflammasome was significantly activated after exposure to LPS and ATP, and this response was reversed by oroxylin A, which was also validated by the reduction of NLRP3 and cleaved-caspase-1 (caspase-1 p10) protein levels and the decrease in IL-1β secretion (Figure 5E and Figure 5F). In addition, oroxylin A significantly inhibited the expressions of NLRP3 and IL-1β at transcription level (Figure 5G). The inhibition on the NLRP3 expression by oroxylin A was also observed in BMDMs without inflammatory stimulation (Figure 5H). Taken together, these data indicated that oroxylin A inhibited NLRP3 inflammasome activation in BMDMs.

### Oroxylin A blocked NLRP3 inflammasome activation through inhibiting the NF-κB signaling

**Figure 6:**
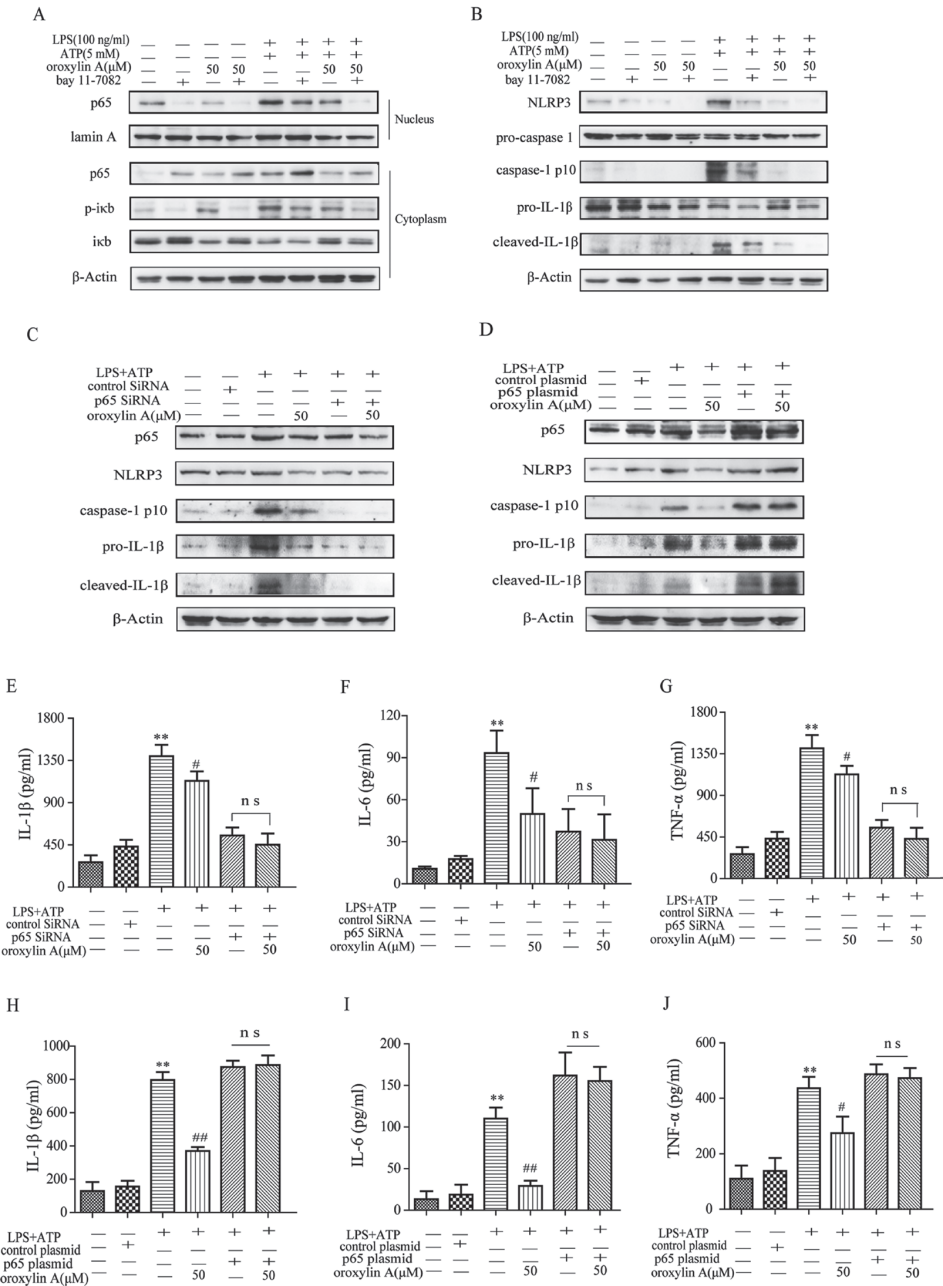
Oroxylin A blocked NLRP3 inflammasome activation through inhibiting the NF-κB signaling (**A**) After pretreatment with bay11-7082 (20 μM), THP-Ms were stimulated with oroxylin A at indicated doses for 12 h. The expressions of cytoplastic protein p65, p-IκB, IκB and nuclear protein NF-κB p65 in THP-Ms were assessed by western blot. (**B**) The expressions of NLRP3, cleaved-caspase-1 (caspase-1 p10) and cleaved-IL-1β proteins were assessed by western blot. (**C**) BMDMs were transfected with NF-κB p65 siRNA for 24 h. Transfected BMDMs were stimulated with LPS and ATP for the indicated time. Protein levels of NLRP3, cleaved-caspase-1 (caspase-1 p10) and cleaved-IL-1β were determined by western Blot. (**D**) BMDMs were transfected with NF-κB p65 overexpression plasmid for 24 h. Transfected BMDMs were stimulated with LPS and ATP for the indicated time. Protein levels of NLRP3, cleaved-caspase-1 (caspase-1 p10) and cleaved-IL-1β were determined by western Blot. (**E**–**G**) BMDMs were transfected with NF-κB p65 siRNA for 24 h. Transfected BMDMs were stimulated with LPS and ATP for the indicated time. IL-1β, IL-6 and TNF-α in supernatant were detected by ELISA. (**H**–**J**) BMDMs were transfected with NF-κB p65 overexpression plasmid for 24 h. Transfected BMDMs were stimulated with LPS and ATP for the indicated time. IL-1β, IL-6 and TNF-α in supernatant were detected by ELISA. Data were shown as means ± SD (*n* = 3). Statistical analysis was performed using one-way ANOVA coupled with a post hoc test. Significant differences were indicated as *P < 0.05 vs. control group (^#^*P* < 0.05 vs. DSS group); **P < 0.01 vs. control group (^##^*P* < 0.01 vs. DSS group).

Next, we investigated the underlying mechanisms which the oroxylin A inhibited NLRP3 inflammasome activation. It is noted that NF-κB activation is required for NLRP3 gene expression and synthesis of pro-IL-1β during the priming step of inflammasome activation [21]. Therefore, we hypothesized that the NF-κB signaling might involve in the inhibitory effect of oroxylin A on NLRP3 inflammasome activation. Indeed, oroxylin A markedly inhibited the increased total NF-κB p65 protein level induced by LPS plus ATP treatment, in a dose-dependent manner (Figure 5I). Meanwhile, the increase in nuclear translocation of NF-κB p65 was dose-dependently decreased by oroxylin A in THP-Ms (Figure 5J and 5L). The production of TNF-α and IL-6, NF-κB-dependent cytokines, was also obviously decreased by oroxylin A, suggesting that oroxylin A inhibited inflammasome activation via NF-κB signaling (Figure 5K). Moreover, bay11-7082, a specific inhibitor of NF-κB, was used to inhibit IκBα activation which could decrease the nuclear translocation of NF-κB p65. Inhibition of NF-κB signaling with bay11-7082 strengthened the inhibitory effects of oroxylin A on NF-κB p65 nuclear translocation and NLRP3 inflammasome activation in THP-Ms, as verified by the reduction of cleaved-caspase-1 (caspase-1 p10), IL-1β and NLRP3 expressions (Figure 6A and 6B). Then, we transfected BMDMs with siRNA targeting NF-κB p65 or p65 overexpression plasmid, and found that NF-κB p65 knockdown greatly reduced NLRP3 inflammasome activation in BMDMs. After transfection, oroxylin A showed little effects on the expressions of cleaved-caspase-1 (caspase-1 p10) and NLRP3 (Figure 6C). And oroxylin A hardly affected the secretion of IL-1β, IL-6 and TNF-α (Figure 6E–6G). In addition, the negative regulation of oroxylin A on NLRP3 inflammasome activation in BMDMs was abolished by NF-κB p65 overexpression plasmid transfection (Figure 6D). Consistent with this, the production of IL-1β, IL-6 and TNF-α was not restrained by oroxylin A after NF-κB p65 plasmid transfection (Figure 6H–6J). These results illustrated that oroxylin A inhibited NLRP3 inflammasome activation through blocking NF-κB signaling.

### Oroxylin A blocked ASC speck formation and inflammasome assembly

**Figure 7:**
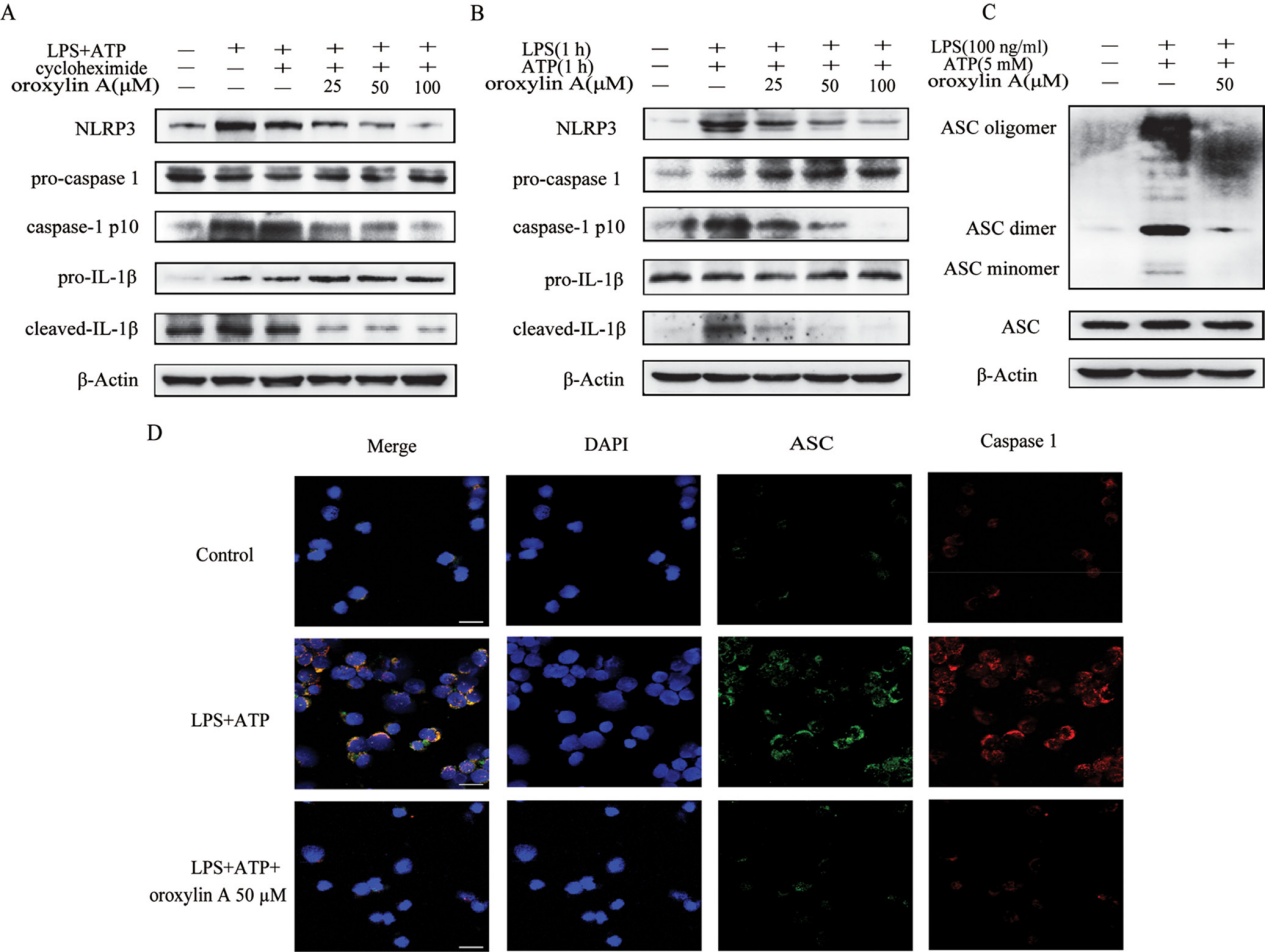
Oroxylin A blocked ASC speck formation and inflammasome assembly (**A**) Oroxylin A-treated THP-Ms were pretreated with cycloheximide for 1 h, and then stimulated with LPS and ATP. Cleaved-caspase-1 (caspase-1 p10), cleaved-IL-1β and NLRP3 were detected by western blot. (**B**) Oroxylin A-treated THP-Ms were stimulated with LPS together with ATP for 1 h. Cleaved-caspase-1 (caspase-1 p10), cleaved-IL-1β and NLRP3 were detected by western blot. (**C**) Immunoblotting of ASC was performed in crosslinked pellets and in cell lysates. (**D**) Immunostaining of endogenous ASC was performed (scale bar, 20 μm). The results are representative of three independent experiments.

The above results demonstrated that oroxylin A could inhibit NLRP3 inflammasome activation via suppression of the priming step. To further elucidate whether oroxylin A could affect the inflammasome formation in the second step, cycloheximide was given before LPS to block NLRP3 protein synthesis. The inhibitory effect of oroxylin A on inflammasome activation was not affected, indicating that oroxylin A could interrupt the inflammasome assembly (Figure 7A). Furthermore, recent studies reported that simultaneous stimulation of LPS and ATP for 1 h triggers rapid caspase-1 cleavage, which is independent of NLRP3 protein level [22], indicating that the synthesis and accumulation of NLRP3 is not essentially involved in rapid caspase-1 activation. As shown in Figure 7B, the cotreatment of THP-Ms with LPS and ATP for 1 h induced rapid NLRP3 inflammasome activation, and oroxylin A treatment had inhibitory effects on it. Otherwise, oroxylin A directly interrupted the ASC speck formation in LPS-primed THP-Ms using IF analysis and Western blot (Figure 7C and 7D). Collectively, these results suggested that oroxylin A inhibited ASC-mediated NLRP3 inflammasome assembly.

## DISCUSSION

Increasing evidences suggest that NLRP3 inflammasome is associated with chronic colitis and colitis-induced colon cancer [4]. NLRP3 is reported to be involved in the identification of pathogens and inflammation-related cytokines production and to play an important role in the body's immune system. The role of NLRP3 in colitis and colitis-induced gastrointestinal tumor has been ambivalent. Zaki et al. reported that NLRP3-deficient mice have a symptom of more exacerbated inflammation than wild-type mice in DSS-induced colitis model, including increased gut barrier damage, immune infiltration and inflammatory cytokines secretion. Besides, Zaki et al. found that the NLRP3-deficient mice are highly susceptible to colitis-induced tumors which associated with increased inflammatory responses and epithelial barrier damages. These processes are ascribed to the decreased production of NLRP3-mediated IL-18 which promotes enterocyte proliferation to repair damaged epithelial cells and maintain intestinal homeostasis [23, 24]. By contrast, Bauer et al. demonstrated that after administration of 2% DSS, NLRP3-deficient mice exhibit lower mortality and attenuated colitis than wild-type mice in acute DSS-induced colitis. This discrepancy may be due to specific composition of the intestinal microbiota and different frequency of CD103+ dendritic cells between NLRP3-deficient and wild-type mice. Bauer et al. further found that after cohousing, wild-type mice are as susceptible as NLRP3-deficient mice, indicating the intestinal microflora plays an important role in the susceptibility towards DSS [8, 12]. In addition, IL-1β, produced mainly by activated NLRP3 inflammasome, can also increase tumorigenesis and promote the migration of cancer cells [25]. Krelin Y et al. found that IL-1β-deficient mice delay chemically induced tumor formation. Likewise, IL-1R antagonist deficient mice, secreting the excessive level of IL-1β, exist rapid tumor development and high tumor frequency. This situation is due to sparse leukocyte infiltrate in IL-1β-deficient mice, whereas a dense neutrophilic infiltrate is found in IL-1Ra–deficient mice [26]. What's more, Voronov E et al. discovered that injection of IL-1β strongly increases metastasis in the murine B16 model, because IL-1β contributes to the production of vascular endothelial cell growth factor in tumor cells [27]. Therefore, NLRP3 inflammasome plays an important role in inflammatory injury repair stage of colitis and migration stage of gastrointestinal tumor.

Recent articles have reported that colitis induced by DSS is mediated by the NLRP3 inflammasome in mice [12]. Thus, DSS-induced acute and chronic colitis models were used here to evaluate the effect of oroxylin A on NLRP3 inflammasome. In this study, oroxylin A ameliorated DSS-induced colonic pathological symptoms and damage as well. Furthermore, we found that oroxylin A decreased MPO and iNOS activities and relieved the inflammatory cells infiltration and colon tissue damage. Moreover, oroxylin A successfully inhibited the production of IL-1β in serum. The mature form of IL-1β requires the activation of caspase-1 which cleaves pro-IL-1β to IL-1β depending on NLRP3 inflammasome [6]. Therefore, we confirmed that oroxylin A could down-regulate the activation of NLRP3 inflammasome, with decreased levels of caspase-1, IL-1β and NLRP3 in colon samples of DSS-induced colitis mice. Besides, no co-housing of NLRP3-/- mice were used to determine the actions of oroxylin A. The results showed that NLRP3-/- mice exhibited a significant improvement of intestinal inflammation induced by DSS, which agreed with Bauer's reports. The reason might be due to the absence of NLRP3, which would greatly interrupt DSS-induced the destruction of epithelial integrity and gut bacterial ecology. Therefore, the deletion of NLRP3 would greatly ameliorate intestinal inflammation. In addition, oroxylin A down-regulated the production of NF-κB downstream targets, including TNF-α and IL-6, in DSS-induced colitis mice. Oroxylin A also dramatically decreased phosphorylated IκB and nuclear translocation of p65 in colon tissues. Taken together, we concluded that oroxylin A inhibited the activation of NLRP3 inflammsome by blocking NF-κB signaling.

In canonical activation models *in vitro*, NLRP3 can form the inflammasome which is sufficient to trigger the activation of the cystein protease caspase-1, and then the activated caspase-1 can convert pro-IL-1β and pro-IL-18 into their mature active forms [6, 20, 28]. NLRP3 inflammasome activation can be divided into two steps. First, treatment of cells with TLR ligand, such as lipopolysaccharide (LPS), bacterial or viral nucleotide, can cause the synthesis of NLRP3 through the NF-κB pathway. The LPS-activated TLR4 recruits the adaptor proteins MyD88 and TRIF to downstream IκB kinase complex (IKK) which activates the NF-κB pathway [19, 29]. Second, the addition of millimolar concentrations of ATP, stimulates the formation of the NLRP3 inflammasome which can play a physiological role in inflammatory diseases [21]. To confirm the results of *in vivo* study, two types of macrophages were used here, and it was found that oroxylin A could down-regulate the activation of NLRP3 inflammasome in both THP-1-derived macrophages and BMDMs. Besides, down-regulation of IL-6 and TNF-α at mRNA level also demonstrated that the inhibition effect of oroxylin A on NLRP3 was associated with NF-κB pathway. Furthermore, we also observed the negative effect of oroxylin A on cytosolic assembly of ASC speck after inflammasome activation, suggesting that oroxylin A could also block the formation of NLRP3 inflammasome.

In conclusion, our results showed that oroxylin A exhibited an inhibitory effect on the activation of NLRP3 inflammasome. This inhibitory effect could be due to the ability of oroxylin A to restrain NF-κB pathway and inflammasome assembly. This study clearly highlighted oroxylin A as a potential agent targeting NLRP3 inflammasome for the clinical treatment of IBD.

## MATERIALS AND METHODS

### Cell culture

Human acute monocytic leukemia THP-1 cells and L929 cells were obtained from Cell Bank of the Chinese Academic of Sciences (Shanghai, China). THP-1 and L929 cells were respectively cultured in RPMI-1640 medium and DMEM, supplemented with 10% fetal bovine serum (Gibco, NY, USA). BMDMs were isolated as previously described [30]. All cell lines were incubated under a humidified 5% (v/v) CO_2_ atmosphere at 37°C.

### Materials

Oroxylin A was isolated from the root of *Scutellaria baicalensis* as previously described [31]. Samples containing oroxylin A at a minimum of 99% purity were used for the experiments unless otherwise indicated. LPS, PMA and ATP were purchased from Sigma Aldrich (St. Louis, MO, USA). DSS (molecular weight 36–50 kDa) was obtained from MP Biomedicals Inc. (Irvine, CA, USA). Antibodies against p-IκBα, IκBα and p65 were purchased from Cell Signaling Technology (Danvers, MA, USA). Primary antibodies against lamin A, β-actin, IL-1β and bay11-7082 were purchased from Bioworld Technology Inc. (CA, USA). Exfect Transfection reagent was purchased from Vazyme (Nanjing, China). NLRP3 and caspase-1 antibodies were from Abcam Technology Inc (MA, USA). FITC-anti-CD11b and FITC-anti-F4/80+ were purchased from eBioscience (San Diego, CA, USA). ELISA kits for murine IL-1β and human IL-1β were purchased from Boster Biotech Co. Ltd. (Wuhan, China). MPO activity assay kit and iNOS Assay Kit were purchased from Nanjing Jiancheng Bioengineering Institute (Nanjing, China). Immunohistochemistry kit was purchased from KeyGEN Biotech Inc. (Nanjing, China).

### Animals

The animal experiment was approved by the Ethics Committee on the Care and Use of Laboratory Animals of China Pharmaceutical University (Nanjing, China). Animal welfare and experimental procedures were carried out strictly in accordance with the Guide for the Care and Use of Laboratory Animals (National Institutes of Health, the United States) and the related ethical regulations of our university. All efforts were made to minimize animals’ suffering and to reduce the number of animals used. 5- to 6-week-old C57BL/6 mice were purchased from Model Animal Genetics Research Center of Nanjing University (Nanjing, China). Pathogen-free male NLRP3-/- C57BL/6 mice were purchased from The Jackson Laboratory.

### Western blot assay

The whole cell lysates were prepared as mentioned [17]. Protein samples were separated by 12% SDS–PAGE and transferred onto the PVDF membranes (Millipore, MA, USA). Then the blots were hybridized with specific antibodies of NLRP3, caspase-1, IL-1β, β-actin and lamin A overnight at 4°C. After washing, blots were exposed to HRP-conjugated secondary antibodies for 1 h at 37°C. All of the antibodies were diluted by PBST with 1% BSA. Enhanced chemoluminescent reagents (Beyotime, Jiangsu, China) were used to detect the proteins on the immunoblots. The protein bands were quantified by using Quantity One software (Bio-rad, CA, USA), the results showed the relative amount of protein that normalized to β-actin.

### Cytoplasmic and nuclear extraction

After treatment, cells were harvested in tubes by centrifugation and washed with ice-cold PBS twice. Nuclear and cytosolic protein extracts were isolated as previously described [15]. Nuclear/Cytosol Fractionation Kit (BioVision, CA, USA) was used according to the manufacturer's instructions. Protein concentrations were quantified with BCA protein assay reagent. Extracts were stored at −20°C until further experimentation.

### ASC oligomerization assay

THP-Ms were seeded in 6-well plates. After the treatment with indicated stimuli, cells were washed by cold PBS and resuspended in an ice-cold buffer (Buffer A: 20 mM HEPES-KOH, pH 7.5, 150 mM KCl, 1% NP-40, 0.1 mM PMSF, and protease inhibitor), and lysed by shearing 10 times through a 21-gauge needle. Nuclei and unlysed cells were removed by centrifugation at 250 g for 5 min. The cell lysates were then centrifuged at 5000 g for 10 min at 4°C. After washing twice with PBS, the pellets were crosslinked with fresh DSS (2 mM) for 30 min at 37°C. The crosslinked pellets were separated in 12% SDS-PAGE and immunoblotting was performed.

### Transfection of NF-κB plasmid and NF-κB siRNA

NF-κB siRNA (Nanjing Rui real biological technology Co. Ltd, Nanjing, china) and NF-κB p65 plasmid (Addgene, Cambridge, MA, USA) were transfected according to manufacturer's instruction of Exfect Transfection reagent (Vazyme, Nanjing, China).

### Immunofluorescence (IF)

CD11b+ and F4/80+ cell infiltration analysis was performed as previously reported [32]. The immunofluorescence assay for NF-κB nuclear translocation was performed according to the method previously described [14, 32]. Briefly, the sections were deparaffinized, rehydrated and washed in 1% PBS-Tween. Then they were treated with 3% hydrogen peroxide for 10 min, blocked with 5% goat serum and incubated with anti-CD11b FITC (1:100) or anti- F4/80+ FITC (1:100) for 1 h at room temperature. The slides were then counter-stained with DAPI for 5 min. Images were observed under a fluorescence microscope (Zeiss, Germany). Settings for image acquisition were identical for control and experimental tissues.

### mRNA extraction and real-time PCR

Total RNA isolation and real-time PCR were performed as previously described [14]. The primer sequences were illustrated as Supplementary Table 1.

### DSS-induced colitis mouse model

The animals were randomised for treatment. Acute colitis model contained 7 days of 2.5% DSS water and 3 days normal water. Oroxylin A was administered through intragastrically daily. C57BL/6 mice were divided into 4 groups randomly: control group, DSS model group, oroxylin A treated groups (100 mg/kg, 200 mg/kg) in acute colitis model. Chronic colitis model contained 3 cycles. Animals received 2.0% DSS for 5 days and then followed by 10 days distilled water in each cycle. C57BL/6 mice were divided into 5 groups randomly: control group, DSS model group, oroxylin A treated groups (50 mg/kg, 100 mg/kg, 200 mg/kg) in chronic colitis model. Oroxylin A was supplemented into the basal diet (AIN-76A, Qinglongshan animal breeding company, Nanjing) and processed by the diet provider. The experimental time lines of the animal model were described in Figure 1B and Figure 3A. The animals were subsequently killed using an overdose of sodium pentobarbital (250 mg/kg, i.p.) and colons were removed, then intestinal tissues were washed with PBS. After measuring the length, the colons were soaked in 10% buffered formalin for 24 h for further histopathological analysis and immunohistochemical assessment.

### Histological analysis

The histological analysis was performed as previously described [17, 33]. Colon tissues were maintained in 10% buffered formalin and embedded in paraffin. Tissue sections were stained with HE to address the degree of inflammation. Histological scoring was determined independently and in a blinded way by a pathologist. Histologic grading score of HE-stained colonic tissues was graded based on following criteria: 0, no inflammatory cells, no signs of inflammation; 1, low leukocyte infiltration, few focal inflammation; 2, moderate leukocyte infiltration, normal crypt epithelium; 3, high leukocyte infiltration, mild crypt epithelium disruption, high vascular density, moderate expansion of the mucosa and focal loss of crypts;

4, severe diffuse inflammation, massive loss of goblet cell and serious crypt epithelium disruption.

### Measurement of MPO and iNOS activity

Neutrophil infiltration into inflamed colonic mucosa was quantified by MPO activity assessment using the O-dianisidine method. Proteins extracted from colonic tissues were used to assess MPO level according to manufacturer's instructions. The supernatant of colonic tissue was measured by Nitric Oxide Synthase Assay Kit according to the manufacturer's recommendations.

### Cytokine analysis by ELISA

Cell supernatants were collected after treatment of oroxylin A, and then determined by ELISA (Boster, Wuhan, China) for IL-1β, IL-6 and TNF-α according to the manufacturer's instructions. Levels of cytokines were expressed in pg/ml.

### Immunohistochemistry

The expressions of NF-κB p65, IL-1β and NLRP3 of the colonic tissues were assessed as described in the previous study [17, 34]. Briefly, after deparaffinization and rehydration, sections were soaked in 3% hydrogen peroxide. Then these slides were treated with 10% goat serum for 2 h at 37 °C. After that, sections were incubated with primary antibodies against NLRP3 and IL-1β in PBS for 2 h at 37 °C. After washing, secondary antibodies were added and incubated at 37 °C for 1 h, and then streptavidin-HRP was added. After 45 min, the slides were stained with DAB Kits and hematoxylin. The immunoreactivity was finally examined under a microscope of Jiangnan XD-202 (Nanjing, China).

### Statistics analysis

The operators and data analysis were blinded. All results shown represent means ± SD from triplicate experiments performed in a parallel manner unless otherwise indicated. Statistical analysis was performed using one-way ANOVA coupled with a post hoc test. See details of each statistical analysis used in the figures and legends.

### Compliance with ethical standards

The animal experiment was approved by the Ethics Committee on the Care and Use of Laboratory Animals of China Pharmaceutical University (Nanjing, China). Animal welfare and experimental procedures were carried out strictly in accordance with the Guide for the Care and Use of Laboratory Animals (National Institutes of Health, the United States) and the related ethical regulations of our university.

## SUPPLEMENTARY MATERIALS TABLE



## References

[1] Ponder A, Long MD (2013). A clinical review of recent findings in the epidemiology of inflammatory bowel disease. Clin Epidemiol.

[2] Podolsky DK (2002). Inflammatory bowel disease. N Engl J Med.

[3] Tontini GE, Vecchi M, Pastorelli L, Neurath MF, Neumann H (2015). Differential diagnosis in inflammatory bowel disease colitis: state of the art and future perspectives. World J Gastroenterol.

[4] Zaki MH, Lamkanfi M, Kanneganti TD (2011). The Nlrp3 inflammasome: contributions to intestinal homeostasis. Trends Immunol.

[5] Biasi F, Leonarduzzi G, Oteiza PI, Poli G (2013). Inflammatory bowel disease: mechanisms, redox considerations, and therapeutic targets. Antioxid Redox Signal.

[6] Jin C, Flavell RA (2010). Molecular mechanism of NLRP3 inflammasome activation. J Clin Immunol.

[7] Villani AC, Lemire M, Fortin G, Louis E, Silverberg MS, Collette C, Baba N, Libioulle C, Belaiche J, Bitton A, Gaudet D, Cohen A, Langelier D (2009). Common variants in the NLRP3 region contribute to Crohn's disease susceptibility. Nat Genet.

[8] Bauer C, Duewell P, Lehr HA, Endres S, Schnurr M (2012). Protective and aggravating effects of Nlrp3 inflammasome activation in IBD models: influence of genetic and environmental factors. Dig Dis.

[9] Guo W, Sun Y, Liu W, Wu X, Guo L, Cai P, Shen Y, Shu Y, Gu Y, Xu Q (2014). Small molecule-driven mitophagy-mediated NLRP3 inflammasome inhibition is responsible for the prevention of colitis-associated cancer. Autophagy.

[10] Kitajima S, Takuma S, Morimoto M (1999). Changes in colonic mucosal permeability in mouse colitis induced with dextran sulfate sodium. Exp Anim.

[11] Liu W, Guo W, Wu J, Luo Q, Tao F, Gu Y, Shen Y, Li J, Tan R, Xu Q, Sun Y (2013). A novel benzo[d]imidazole derivate prevents the development of dextran sulfate sodium-induced murine experimental colitis via inhibition of NLRP3 inflammasome. Biochem Pharmacol.

[12] Bauer C, Duewell P, Mayer C, Lehr HA, Fitzgerald KA, Dauer M, Tschopp J, Endres S, Latz E, Schnurr M (2010). Colitis induced in mice with dextran sulfate sodium (DSS) is mediated by the NLRP3 inflammasome. Gut.

[13] Tseng TL, Chen MF, Tsai MJ, Hsu YH, Chen CP, Lee TJ (2012). Oroxylin-A rescues LPS-induced acute lung injury via regulation of NF-kappaB signaling pathway in rodents. PLoS One.

[14] Yao J, Hu R, Sun J, Lin B, Zhao L, Sha Y, Zhu B, You QD, Yan T, Guo QL (2014). Oroxylin A prevents inflammation-related tumor through down-regulation of inflammatory gene expression by inhibiting NF-kappaB signaling. Mol Carcinog.

[15] Ye M, Wang Q, Zhang W, Li Z, Wang Y, Hu R (2014). Oroxylin A exerts anti-inflammatory activity on lipopolysaccharide-induced mouse macrophage via Nrf2/ARE activation. Biochem Cell Biol.

[16] Chen Y, Yang L, Lee TJ (2000). Oroxylin A inhibition of lipopolysaccharide-induced iNOS and COX-2 gene expression via suppression of nuclear factor-kappaB activation. Biochem Pharmacol.

[17] Yang X, Zhang F, Wang Y, Cai M, Wang Q, Guo Q, Li Z, Hu R (2013). Oroxylin A inhibits colitis-associated carcinogenesis through modulating the IL-6/STAT3 signaling pathway. Inflamm Bowel Dis.

[18] Lorenz RG, McCracken VJ, Elson CO (2005). Animal models of intestinal inflammation: ineffective communication between coalition members. Springer Semin Immunopathol.

[19] Bauernfeind FG, Horvath G, Stutz A, Alnemri ES, MacDonald K, Speert D, Fernandes-Alnemri T, Wu J, Monks BG, Fitzgerald KA, Hornung V, Latz E (2009). Cutting edge: NF-kappaB activating pattern recognition and cytokine receptors license NLRP3 inflammasome activation by regulating NLRP3 expression. J Immunol.

[20] Lamkanfi M, Kanneganti TD (2010). Nlrp3: an immune sensor of cellular stress and infection. Int J Biochem Cell Biol.

[21] Kanneganti TD, Lamkanfi M, Nunez G (2007). Intracellular NOD-like receptors in host defense and disease. Immunity.

[22] Lin KM, Hu W, Troutman TD, Jennings M, Brewer T, Li X, Nanda S, Cohen P, Thomas JA, Pasare C (2014). IRAK-1 bypasses priming and directly links TLRs to rapid NLRP3 inflammasome activation. Proc Natl Acad Sci USA.

[23] Zaki MH, Boyd KL, Vogel P, Kastan MB, Lamkanfi M, Kanneganti TD (2010). The NLRP3 inflammasome protects against loss of epithelial integrity and mortality during experimental colitis. Immunity.

[24] Zaki MH, Vogel P, Body-Malapel M, Lamkanfi M, Kanneganti TD (2010). IL-18 production downstream of the Nlrp3 inflammasome confers protection against colorectal tumor formation. J Immunol.

[25] Klampfer L (2011). Cytokines, inflammation and colon cancer. Curr Cancer Drug Targets.

[26] Krelin Y, Voronov E, Dotan S, Elkabets M, Reich E, Fogel M, Huszar M, Iwakura Y, Segal S, Dinarello CA, Apte RN (2007). Interleukin-1beta-driven inflammation promotes the development and invasiveness of chemical carcinogen-induced tumors. Cancer Res.

[27] Voronov E, Shouval DS, Krelin Y, Cagnano E, Benharroch D, Iwakura Y, Dinarello CA, Apte RN (2003). IL-1 is required for tumor invasiveness and angiogenesis. Proc Natl Acad Sci USA.

[28] Lalor SJ, Dungan LS, Sutton CE, Basdeo SA, Fletcher JM, Mills KH (2011). Caspase-1-processed cytokines IL-1beta and IL-18 promote IL-17 production by gammadelta and CD4 T cells that mediate autoimmunity. J Immunol.

[29] Rahman MM, McFadden G (2011). Myxoma virus lacking the pyrin-like protein M013 is sensed in human myeloid cells by both NLRP3 and multiple Toll-like receptors, which independently activate the inflammasome and NF-kappaB innate response pathways. J Virol.

[30] Liu X, Zhang X, Ding Y, Zhou W, Tao L, Lu P, Wang Y, Hu R (2017). Nuclear Factor E2-Related Factor-2 Negatively Regulates NLRP3 Inflammasome Activity by Inhibiting Reactive Oxygen Species-Induced NLRP3 Priming. Antioxid Redox Signal.

[31] Li HB, Chen F (2005). Isolation and purification of baicalein, wogonin and oroxylin A from the medicinal plant Scutellaria baicalensis by high-speed counter-current chromatography. J Chromatogr A.

[32] Sun Y, Zhao Y, Yao J, Zhao L, Wu Z, Wang Y, Pan D, Miao H, Guo Q, Lu N (2015). Wogonoside protects against dextran sulfate sodium-induced experimental colitis in mice by inhibiting NF-kappaB and NLRP3 inflammasome activation. Biochem Pharmacol.

[33] Lowe EL, Crother TR, Rabizadeh S, Hu B, Wang H, Chen S, Shimada K, Wong MH, Michelsen KS, Arditi M (2010). Toll-like receptor 2 signaling protects mice from tumor development in a mouse model of colitis-induced cancer. PLoS One.

[34] Yao J, Pan D, Zhao Y, Zhao L, Sun J, Wang Y, You QD, Xi T, Guo QL, Lu N (2014). Wogonin prevents lipopolysaccharide-induced acute lung injury and inflammation in mice via peroxisome proliferator-activated receptor gamma-mediated attenuation of the nuclear factor-kappaB pathway. Immunology.

