# Supplementary material for: Oroxylin A inhibits colitis by inactivating NLRP3 inflammasome
Source: Oncotarget. 2017 Jul 22;8(35):58903–17. doi: 10.18632/oncotarget.19440 (PMC5601702; doi:10.18632/oncotarget.19440)
Supplement: Supplementary file 1 [file oncotarget-08-58903-s001.pdf]

Oroxylin A inhibits colitis by inactivating NLRP3 inflammasome

SUPPLEMENTARY MATERIALS

Supplementary Table 1: Details of primer sequences used

Human:

	Forward	Reverse
IL-1β	5′-AGGCTGCTCTGGGATTC-3′	5′-GCCACAACAACTGACGC-3′
NLRP3	5′-AACATTCGGAGATTGTGGTTGGG-3′	5′-GTGCGTGAGATTCTGATTAGTGCTG-3
IL-6	5′-GACAAACAAATTCGGTACATCCTCG-3′	5′-GGGTCAGGGGTGGTTATTGC-3′
TNF-α	5′-CTCTCACATACTGACCCACGGCT-3′	5′-GGGTTCGAGAAGATGATCTGACTGC-3′
GAPDH	5′-AAGGTCGGAGTCAACGGATTT-3′	5′-AGATGATGACCCTTTTGGCTC-3′

Mouse:

	Forward	Reverse
IL-1β	5′-TCATTGTGGCTGTGGAGAAG -3′	5′-AGGCCACAGGTATTTTGTCG -3′
NLRP3	5′-TGCAGAAGACTGACGTCTCC -3′	5′-CGTACAGGCAGTAGAACAGTTC -3′
IL-6	5′-GACAAACAAATTCGGTACATCCTCG-3′	5′-GCATTGGAAATTGGGGTAGGAA-3′
TNF-α	5′-CGAGTGACAAGCCTGTAGCCC-3′	5′-GTCTTTGAGATCCATGCCGTTG-3′
GAPDH	5′-TCAACGGCACAGTCAAGG-3′	5′- ACCAGTGGATGCAGGGAT -3′
